# Clinical profile of venomous snake bites in north Indian Military Hospital

**DOI:** 10.4103/0974-2700.43184

**Published:** 2008

**Authors:** Jasjit Singh, Sanjeev Bhoi, Vineet Gupta, Ashish Goel

**Affiliations:** 1 Department of Medicine, Military Hospital, Amritsar; 2 Division of Emergency Medicine, JPNATC, All India Institute of Medical Sciences, New Delhi – 110 029, India

**Keywords:** Anti-snake venom, neurotoxicity, snakebite

## Abstract

Snakebite is an environmental hazard associated with significant morbidity and mortality. We report a case series of venomous snakebites in a military operational area of north India. Of 33 cases of snake bites presenting to the military hospital, 21 patients were envenomated. The median age of patients was 24 years; all were men. All of the envenomations were neurotoxic in nature. Abdominal pain (91%), headache (86%), dysphagia (86%), ptosis (77%), diplopia (72%), blurred vision (72%), dyspnea (67%), and vomiting (62%) were the predominant clinical presentation. Polyvalent AntiSnakeVenom (ASV) [mean 180 ml; range 90-320 ml] was given to all patients with systemic manifestations, and repeated as needed. Eleven (52%) patients received neostigmine with glycopyrrolate to counter cholinergic effects. Two patients were given ventilatory support. The average time of recovery from envenomation was 16 hours after administration of ASV. All patients recovered without sequelae. Soldiers during military exercise are vulnerable to snakebites. Neurotoxic snakebites predominate in our study and usually present with autonomic features along with headache, abdominal pain, ptosis, diplopia and dysphasia. Preventive measures to minimize snake bites and planned treatment regimens should be emphasized among medical and military personnel deployed in the field operations.

## INTRODUCTION

Snakebite is an environmental hazard associated with significant morbidity and mortality. In Asia alone, snakebites are estimated at about four million cases per year, of which approximately 50% are envenomations, with about 100,000 annual deaths. In India, an estimated 200,000 persons per year fall prey to snake-bite, with an estimated fatality rate of 35,000-50,000 per year.[1] Delayed presentation to hospitals frequently contributes to increased morbidity and mortality from snakebites. The delay is due to people using folk and indigenous remedies before reaching the hospital especially in rural areas. The incidence of envenomation is particularly high in tropical regions where snakes are abundant and human activities like field work and sleeping outdoors increase the risk of man-snake encounters.[2] Snake bites may be a serious occupational hazard for the military personnel deployed in snake prone areas. The troops are exposed to the natural habitat of snakes during such military training exercises. A few studies from developed nations have reported incidence of snake bites among the soldiers in field.[3–5] Since there is no Indian data on Snakebite in Military population in India we decided to study the issue as a occupational hazard in soldiers.

## MATERIALS AND METHODS

The study is a descriptive case series. Prospective data was collected. It was conducted during the “Operation Parakram” that included field deployment of military personnel along the Western border of North India during June to October in 2002. All soldiers reporting to the emergency department (ED) of military hospital in Amritsar, India, with alleged history of snakebite were included in the study after they consented to participate. Exclusion criteria were an ambiguous history, a non-venomous or “dry” bite, or a scorpion or spider had caused the bite. Non-venomous bites were defined by a lack of signs or symptoms of snakebite after a period of observation. Patients in who the initial symptoms were transient and consistent with a panic reaction due to the sighting of the snake were also excluded from the study. Venomous bites were defined by the presence of signs and symptoms of local and/or systemic toxicity. Local toxicity was defined by the presence of a local reaction in the form of swelling, bleeding from fang marks, cellulitis, or necrosis. Signs and symptoms of toxicity were either neuroparalytic or hemostatic abnormalities such as bleeding from mucocutaneous sites, systemic bleeding, intravascular hemolysis, or a deranged laboratory coagulation profile anytime during hospital stay. A neuroparalytic syndrome included sensory or motor paralysis in the form of paresthesias, taste and smell abnormalities, ptosis, cranial nerve palsy, general flaccidity, or respiratory paralysis. Recovery from envenomation was defined by the resolution of local and systemic signs clinically and/or on laboratory investigations. Polyvalent anti snake venom (ASV) and other supportive therapy were instituted as per hospital's protocol. ASV was repeated depending on the clinical response to therapy determined by alleviation of systemic manifestations. The time to onset of symptoms, presentation to hospital, clinical examination, serial management including the amount of ASV administered, and any adverse event were recorded. Descriptive statistical analysis was performed using SPSS software, version 10.

## RESULTS

A total of 33 cases of alleged snake bites reporting to the hospital between June and October 2002 were included in the study. 12 of the 33 (33.3%) cases were non venomous and were excluded from subsequent analysis. The median age was 24 years (range 18- 38 years); all patients were men. 81% of bites were in the lower third of leg and feet; three (14%) were bitten on thigh and buttocks primarily while defecating in open fields. Three patients were bitten on fingers while cutting grass. Fang marks were not found in two cases due to swelling. Three patients brought dead snakes to the ED, which were identified as kraits. A tourniquet was applied in seven cases before presentation to the hospital. All patients were anxious and agitated. All bites were neurotoxic in nature manifesting as neuroparalytic syndrome. The time to onset of symptoms was between 30 and 60 minutes, while the time to report to the ED ranged between 30 and 180 minutes after the bite. Abdominal pain (91%), headache (86%), dysphagia (86%), ptosis (77%), diplopia (72%), blurring of vision (72%), dyspnea (67%) vomiting (62%), were the predominant clinical presentation. Lassitude (48%), perioral paresthesia (48%), respiratory paralysis (10%) and dryness of mouth (10%) were the less frequently observed symptoms. Leucocytosis and azotemia were reported in five patients. The azotemia was pre-renal and this was attributed secondary to recurrent vomiting. Two patients needed ventilatory support for overt respiratory paralysis.

All 33 cases were hospitalized. Non-venomous bites (n=12) were discharged after 48-72 hours of hospitalization. The median duration of hospitalization for venomous bite cases was 4 days (range 3-13 days). The median dose of ASV administered to all symptomatic patients was 180 ml (range 90-320 ml). One patient developed anaphylaxis and two developed mild pyrexial reaction to ASV. 11 (52%) patients received neostigmine with glycopyrrolate to counter cholinergic effects. The median time of recovery from envenomation was 8 hours (range 6-120 hours) after initiating ASV. All patients recovered without any sequelae.

## DISCUSSION

Snake bite is a common medical emergency and an occupational hazard especially in tropical India. In the present case series the patients were young male soldiers posted in field areas for military exercise. In the current study patients were bitten during unprovoked encounters at night while asleep in tents or bunkers; or early morning during open field sanitation. Shiau *et al.* indicated association of poor toileting facilities (trench toilets) with increased incidents of snake bites. They suggested increased risk associated with the more temporary types of field sanitation infrastructure as a surrogate measure for troops being exposed to arthropods and snakes.[5] Low cost mobile toilets, building snake pits around designated sanitation area, keeping the toilet area clean of any possible hideouts for snakes could help minimize snakebite incidence in troops.

Most of the patients in our series presented to the hospital early, which is in disagreement with some of the other studies where a later presentation has been reported.[6,7] This earlier presentation could be due to increased anxiety and fright in the afflicted subjects and better access to transportation available to the soldiers.

We did not observe the use of herbal medicine or traditional remedies like incision of the wound in our patients, but tight tourniquets were applied in one third cases. This signifies a lack of popular awareness about current prehospital management recommendations for snake bites. Definite protocol for field treatment of snakebites like that devised by the Israeli defense forces needs to be considered for the Indian setup too.[5] Recommended field management includes reassurance, splinting, rest, intravenous fluid administration, and pain medication followed by rapid evacuation to a hospital for definitive care and antivenin administration. Contraindicated measures include cutting or applying suction to the wound, applying arterial or venous tourniquets, giving hot fluids or alcohol, cooling the wound, or cauterizing or freezing the wound.[5]

Punde *et al.* reported 633 snake bites in farmers from rural Maharashtra most of whom were young males and presented with neurotoxic symptoms.[8] In another study form the same area, Bawaskar *et al.* also found primarily neurotoxic manifestations of snakebite in the form of ptosis, respiratory muscle weakness, ophthalmoplegia and limb weakness, and showed a mortality rate of 5.4%.[9] Neurotoxicity also was the predominant manifestation of snakebite in the present study. Headache, dyphagia, ptosis, diplopia were predominant neurotoxic manifestations observed. We report only two patients (10%) needing ventilatory support in contrast to other studies with one fourth to three fourth of neurotoxic bites needing assisted ventilation.[7,10] Usually neurotoxicity occurs within 60 minutes of envenomation, rapidly progressing to respiratory paralysis requiring early ventilatory support.[1] There was no mortality among the patients in the current study. Earlier studies have reported mortality rates between 5% and 10%.[6–9] Early reporting to hospital attributed to availability of an on-field nursing assistant, better access to transportation facilities for soldiers, and prompt institution of definitive therapy (ASV and supportive treatment) may have contributed to low mortality in the present study.

In the present series, the mean ASV used was 180 ml (range 90-320 ml). This falls on the higher range for ASV dosage. Vijeth *et al.* have reported similar mean effective dose of ASV while lower doses have been found effective by Tariang *et al.* (47 ml) and Paul *et al.* (60 ml).[11,12] Currently lack of definitive guideline regarding the optimum dosage has prompted physicians to use ASV empirically in higher doses. Judicious use of ASV should be encouraged in physicians especially in the field to avoid any adverse effects due to its overdose.

Prevention remains the best method to reduce man-snake encounters. Murdoch *et al.* and Krysa-clark *et al.* emphasized the importance of prevention and emergency field management of venomous snakebites during military exercises.[3,4] Measures like wearing protective clothing and shaking clothes/shoes before wearing are useful activities in this regard. Soldiers should avoid disturbing animal habitats or provoking the snakes.[5] Awareness of the seasonal variations along with local landscape study can also help minimize such human snake encounters.

## CONCLUSION

In conclusion, soldiers during military exercise are vulnerable to snakebites. Neurotoxic snakebites predominated in our study and usually presented with autonomic features along with headache, ptosis, diplopia and dysphasia. These symptoms may be subject to wide regional variations as evident in literature. Early recognition of snakebite, prompt institution of polyvalent antisnake venom as well as respiratory support are prudent in preventing mortality. A definite prehospital field plan, improved sanitation and awareness promotion for snakebites during military exercise would not only reduce mortality following a snakebite but also reduce the incidence of such encounters.

## RESEARCH DETAILS

The study was conducted at Military Hospital Amritsar and the analysis was done at All India Institute of Medical Sciences. Ethical Clearance was taken at Hospital Ethics committee of Military Hospital Amritsar. Since there is no Indian data on Snakebite in Military population in India we decided to study the issue as a occupational hazard in soldiers.
